# Supplementary material for: Out-of-pocket expenditure on medicines in Bangladesh: An analysis of the national household income and expenditure survey 2016–17
Source: PLoS One. 2022 Sep 16;17(9):e0274671. doi: 10.1371/journal.pone.0274671 (PMC9480983; doi:10.1371/journal.pone.0274671)
Supplement: S1 File — HIES, Bangladesh, 2016. (DOCX) [file pone.0274671.s001.docx]

| **Annex 1.** Adjusted incremental risk of OOPE on medicines according to household presence chronic diseae. HIES, Bangladesh, 2016 | | | | |
| --- | --- | --- | --- | --- |
|  |  |  |  |  |
|  | OOPE on medicines > 0 | Share of OOPE on medicines out-off OOPE on health | OOPE on medicines per adult equivalent (ln) | Share of OOPE on medicines out-off household expenditure |
| Any member with a chronic disease | 1.26 [1.17―1.35] | 1.39 [1.32―1.45] | 1.06 [1.01―1.12] | 1.82 [1.62―2.01] |
| Often infectious origin | 1.10 [1.06―1.13] | 0.98 [0.96―1.01] | 1.02 [0.99―1.06] | 0.99 [0.92―1.06] |
| Disabilities | 1.07 [1.04―1.10] | 0.99 [0.97―1.04] | 1.08 [1.05―1.11] | 1.11 [1.04―1.17] |
| Diabetes | 1.09 [1.04―1.13] | 1.01 [0.97―1.04] | 1.24 [1.20―1.27] | 1.19 [1.11―1.28] |
| Cardiovascular disease | 1.11 [1.08―1.16] | 1.02 [0.99―1.05] | 1.16 [1.12―1.20] | 1.17 [1.09―1.24] |
| Cancer | 1.08 [1.00―1.16] | 0.85 [0.74―0.97] | 1.60 [1.45―1.76] | 2.32 [1.63―3.01] |
| Others chronic disease | 1.09 [1.06―1.11] | 0.98 [0.95―0.99] | 1.26 [1.23―1.29] | 1.44 [1.36―1.52] |
