# Supplementary material for: Out-of-pocket expenditure on medicines in Bangladesh: An analysis of the national household income and expenditure survey 2016–17
Source: PLoS One. 2022 Sep 16;17(9):e0274671. doi: 10.1371/journal.pone.0274671 (PMC9480983; doi:10.1371/journal.pone.0274671)
Supplement: Attachment — Submitted filename: Responses to the editors and reviewers comments_August 7.pdf [file pone.0274671.s004.pdf]

Responses to the reviewer's comments:

Dear Editor,
We would like to thank you for your response to our letter to the Editor on July 26 (see the Annex to this letter). **You asked us to edit the language**. In response to this request, we have significantly edited the language. You will see these changes in the **track-change version** that we uploaded.

Additional responses to the reviewer's comments can be found in our letter to the Editor on July 15, 2022 (also in the Annex).

Annex: Recent correspondence with PLoS

From: plosone <plosone@plos.org>
Sent: Tuesday, July 26, 2022 5:49 PM
To: Wirtz, Veronika <vwirtz@bu.edu>
Subject: RE: PLOS ONE Decision: Revision required [PONE-D-21-14254R3]

Dear Dr. Wirtz,

Thank you for your patience, here is the response of the AE regarding to your concern in reviewers comment.

******
Thanks for your email. I have gone through the comments and responses. I think the authors are valid in their argument. But they still need to edit the language. Please ask them to do so, and then I will provide my final decision.
****

Feel free to message me if you have any further question or concern

Kind regards,

Liezl Callo
Straive Editorial Assistant

PLOS ONE | plosone@plos.org
Empowering researchers to transform science

Boston, July 15,2022

Dear Editor
We are responding to the most recent request for changes to this manuscript. Respectfully, we would like to ask that the Journal make a final determination as to whether or not our manuscript [PONE-D-21-14254R3] - [EMID:e4ab9e939629ff6c] is acceptable for publication without making any additional changes. One of the reviewers, Ajantha Sisira Kumara, keeps insisting (now for the third time) that we provide two additional analyses. In our view, these analyses are both out of the scope of our study and, in fact, are not feasible. We have explained our reasoning in our detailed responses over the past three re-submissions, which can be found below. Indeed, we believe these requests would amount to writing a separate paper.

It is noteworthy that all other reviewers over the course of the three revisions have been satisfied with our responses and how we incorporated their comments except Ajantha Sisira Kumara who keeps coming back with the same request over and over regardless of our explanation.

Since it is clear from Kumara's comments that nothing is wrong with the current manuscript and that this request for additional analyses is complementary and not essential, we respectfully ask the Journal to please accept the manuscript at this stage.

Sincerely,

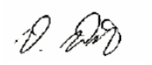

Veronika J. Wirtz, B.Pharm., M.Sc., Ph.D., FISPE
Professor - Department of Global Health, Boston University School of Public Health
Director, World Health Organization Collaborating Center in Pharmaceutical Policy

vwirtz@bu.edu; T: +1 617 358 3046
https://www.bu.edu/sph/profile/veronika-wirtz/
pronouns: she/her/hers @VeroWirtz

1st Revision October 2021

Ajantha Sisira Kumara: "The importance of supply-side factors in determining OOP expenses for medicines is completely ignored from the study, making it lopsided. Do you have data in HIES on healthcare supply-side factors? For instance, distance to the nearest government /private hospital, nearest public/private clinics, nearest public/private dispensary, and availability of doctors, specialists, dentists per 1,000 of population as they might have an explanatory power. My recommendation would be incorporate such variables and see whether they play a role in Bangladesh like in other contexts."

RESPONSE: The purpose of this study is to analyze household and individual factors that are associated with medicines OOPE. The reviewer is correct that additional data on the supply side could be added to complete this analysis. However, the survey does not provide geospatial data at the unit of analysis which is the household. Adding supply side factors such as nearest public dispensary, etc. would be approximations based on the smallest geographical area. We have added in the discussion a section where we expand on the need for analysis of the supply-side factors.

"Finally, this study focuses on determinants of out-of-pocket expenditure that are recorded within this survey. We did not link other databases to study determinants of expenditure that are not included in the survey such as distance to the nearest government /private hospital, nearest public/private clinics, nearest public/private dispensary, and availability of doctors, specialists, dentists per 1,000 of population as they might have an explanatory power."

2nd Revision in December 2022

All comments successfully responded to.

3rd Revision May 2022

Ajantha Sisira Kumara: "Please check whether you could attend the followings which may further improve your paper: • From the viewpoint of public policy, it may be important to discuss about income elasticity of OOP expenses on medicine. Can you calculate the elasticity using double-log regression framework? Analyzing the data from elasticity ground may be more important than analyzing the same across different income quintiles. This type of an analysis may be useful to determine whether medicine is an essential or a luxury for Bangladeshi households."

RESPONSE: As the reviewer suggests we have incorporated this aspect in the discussion section.

“Sixth, this study does not analyze medicines prices or price elasticity as a factor affecting medicines out-of-pocket expenditure because information on medicine prices is not collected as part of the survey. Linking outside data source with the data set used in this study is challenging as individuals in the survey do not report on specific products that they purchased.”

Moreover, it is noteworthy to mention to the reviewer that medicine price information collected through market studies often do not reflect the prices patients pay for their medicines.

Ajantha Sisira Kumara: “The covariates that the paper considered represent only the demand-side of medicines. The paper misses the story coming from supply-side factors in determining OOP expenses on medicines. Availability of public/private avenues for treatments, proximity to such avenues, health insurance coverage, facilities available in public/private avenues, influence of doctors are some of supply-side factors. Can you strengthen the analysis by incorporating the information pertaining to those supply-side factors? Accordingly, you may strengthen the sections of discussions and conclusions by describing the situation and the role of such supply-side factors in determining OOP expenses on medicines.”

RESPONSE: The reviewer is correct that this analysis focuses on the demand-side. It would be very interesting to link the demand side data with the supply side. However, this is outside the scope of this manuscript. In addition, it is noteworthy, that linking supply side factors such as availability of medicines in public and private sector and prescriber behavior in the catchment area of individual households are often absent or not updated. We have included this limitation in the discussion sector.

“Finally, this study focuses on determinants of out-of-pocket expenditure that are recorded within this survey. We did not link other databases to study determinants of expenditure such as distance to the nearest government/private hospital, nearest public/private clinics, nearest public/private dispensary, and availability of doctors, specialists, dentists per 1,000 of population as they might have an explanatory power.”

4th Revision July 2022

Ajantha Sisira Kumara: “However, I still believe that you can calculate income elasticity of medical expenditure at the household-level. For this purpose, you no need to have the data pertaining to unit prices of medicine. I hope your dataset has household income or otherwise, it can be proxied by household expenditure.

Further, I still believe that you can incorporate health-related supply-side factors to calculate their impact on OOP expenditure on medicine. Those data are available at the country's macro-level and can be collected from annual reports of relevant ministries.

I firmly believe that the paper will be in a better shape after you address at least these two issues. Congratulations!”
