# Supplementary material for: Out-of-pocket expenditure on medicines in Bangladesh: An analysis of the national household income and expenditure survey 2016–17
Source: PLoS One. 2022 Sep 16;17(9):e0274671. doi: 10.1371/journal.pone.0274671 (PMC9480983; doi:10.1371/journal.pone.0274671)
Supplement: Attachment — Submitted filename: Response to Reviewers .docx [file pone.0274671.s002.docx]

PONE-D-21-14254R1

**Out-of-pocket expenditure on medicines in Bangladesh: an analysis of the national household income and expenditure survey 2016-17**

PLOS ONE

We would like to thank the reviewer for the comment. We have responded to it below and made the requested changes in the text of the manuscript (marked in **bold**).

Reviewer #2: While you have estimated the catastrophic health spending using capacity to pay approach, you have not estimated the estimates using threshold of 40%. In CTP approach a single threshold of 40% is used. If you will do so, you will find different estimates by quintile. Your revised table 4 is not correct. It is based on budget share approach Pl read the following reference to get complete idea of estimating CHE using CTP approach

https://equityhealthj.biomedcentral.com/articles/10.1186/s12939-021-01421-6

Addressing data and methodological limitations in estimating catastrophic health spending and impoverishment in India, 2004–18

**Authors’ response: Thank you very much for your comment. In response to your suggestion and following the article you shared with us, we disaggregated Table 4 into two separate tables (see below), the first (Table 4) for the adjusted ratio of spending on medicines with respect to total household spending, and the second (Table 5) for the adjusted ratio of spending on medicines with respect to of households' ability to pay. In Table 5 we have the additional threshold of 40% of the households' ability to pay. We clarify this point in the methods section as follows:**

*[…]* *Finally, based on the first regression analysis results, we estimated the adjusted share of OOPE on medicines out of total household expenditure* ***(considering the following thresholds of the total household expenditure: 10, 15, 20, 30%); and*** *out of a household’s capacity to pay by considering different thresholds (10, 15, 20, 30* ***and 40%*** *of the total household expenditure)* ***[31]*** *and according to the quintile of household expenditure per equivalent adult […]*

**We have added further descriptions of the results:**

*“The disparity between the lowest and wealthiest households increases with higher thresholds: while 0.5% of household spent over 40% of their disponible household income on medicines, only 0.2% of the wealthiest households do so.”*

Reference: 31. Mohanty, S.K., Dwivedi, L.K. Addressing data and methodological limitations in estimating catastrophic health spending and impoverishment in India, 2004–18. Int J Equity Health 20, 85 (2021). https://doi.org/10.1186/s12939-021-01421-6

**We have included the revised Table 4 and a new Table 5 in the manuscript.**

Table 4. Adjusted share of OOPE on medicines out-of total household expenditure according to quintile of household expenditure. HIES, Bangladesh, 2016/17

| Threshold | Overall | Quintile of monthly household expenditure per equivalent adult | | | | |
| --- | --- | --- | --- | --- | --- | --- |
|  |  | 1st | 2nd | 3th | 4th | 5th |
| ≥10% | 26.178 [25.003―27.353] | 27.279 [25.475―29.083] | 26.246 [24.583―27.909] | 26.442 [24.485―28.398] | 27.525 [25.524―29.527] | 23.614 [21.276―25.952] |
| ≥15% | 6.820 [6.362―7.278] | 6.649 [5.809―7.488] | 6.760 [5.991―7.529] | 7.200 [6.241―8.160] | 7.113 [6.255―7.972] | 6.396 [5.429―7.363] |
| ≥20% | 1.824 [1.619―2.028] | 1.746 [1.390―2.102] | 1.725 [1.370―2.080] | 1.911 [1.399―2.423] | 1.910 [1.488―2.333] | 1.825 [1.402―2.249] |
| ≥30% | 0.138 [0.095―0.181] | 0.124 [0.038―0.210] | 0.078 [0.007―0.149] | 0.200 [0.068―0.333] | 0.128 [0.046―0.210] | 0.159 [0.058―0.260] |

Table 5. Adjusted share of OOPE on medicines out-of household’s capacity to pay according to quintile of household expenditure. HIES, Bangladesh, 2016/17

| Threshold | Overall | Quintile of monthly household expenditure per equivalent adult | | | | |
| --- | --- | --- | --- | --- | --- | --- |
|  |  | 1st | 2nd | 3th | 4th | 5th |
| ≥10% | 71.058 [69.262―72.854] | 80.376 [78.767―81.984] | 78.532 [76.635―80.429] | 74.696 [72.274―77.118] | 69.612 [66.928―72.296] | 53.394 [49.288―57.500] |
| ≥15% | 42.213 [40.557―43.869] | 52.660 [50.628―54.691] | 48.027 [45.742―50.312] | 43.939 [41.495―46.383] | 40.161 [37.592―42.729] | 27.519 [24.531―30.508] |
| ≥20% | 18.547 [17.568―19.525] | 23.816 [22.170―25.463] | 20.960 [19.493―22.427] | 19.501 [17.748―21.253] | 16.957 [15.380―18.534] | 12.078 [10.518―13.637] |
| ≥30% | 2.760 [2.503―3.016] | 3.458 [2.796―4.120] | 3.114 [2.594―3.633] | 2.806 [2.197―3.414] | 2.613 [2.118―3.108] | 1.885 [1.460―2.310] |
| ≥40% | 0.296 [0.230―0.363] | 0.527 [0.329―0.725] | 0.268 [0.126―0.412] | 0.250 [0.099―0.401] | 0.250 [0.120―0.380] | 0.206 [0.092―0.320] |
