# Supplementary material for: Out-of-pocket expenditure on medicines in Bangladesh: An analysis of the national household income and expenditure survey 2016–17
Source: PLoS One. 2022 Sep 16;17(9):e0274671. doi: 10.1371/journal.pone.0274671 (PMC9480983; doi:10.1371/journal.pone.0274671)
Supplement: Attachment — Submitted filename: Responses_PLoS One Bangladesh OOP medicines_Final.docx [file pone.0274671.s003.docx]

**Responses to the reviewers' comments**

**Manuscript [PONE-D-21-14254R1]**

We would like to thank the reviewers for the constructive comments. We have carefully addressed the each of the reviewer’s comments in this revision. We believe that after incorporating your thoughtful feedback, our manuscript has been improved. Our responses to the reviewer’s comment can be found in bold.

1. If the authors have adequately addressed your comments raised in a previous round of review and you feel that this manuscript is now acceptable for publication, you may indicate that here to bypass the “Comments to the Author” section, enter your conflict of interest statement in the “Confidential to Editor” section, and submit your "Accept" recommendation.

Reviewer #1: All comments have been addressed

Reviewer #2: All comments have been addressed

**RESPONSE: Thank you.**

2. Is the manuscript technically sound, and do the data support the conclusions?

The manuscript must describe a technically sound piece of scientific research with data that supports the conclusions. Experiments must have been conducted rigorously, with appropriate controls, replication, and sample sizes. The conclusions must be drawn appropriately based on the data presented.

Reviewer #1: Partly

Reviewer #2: Yes

**RESPONSE: Thank you.**

3. Has the statistical analysis been performed appropriately and rigorously?

Reviewer #1: Yes

Reviewer #2: Yes

**RESPONSE: Thank you.**

4. Have the authors made all data underlying the findings in their manuscript fully available?

The [PLOS Data policy](http://www.plosone.org/static/policies.action#sharing) requires authors to make all data underlying the findings described in their manuscript fully available without restriction, with rare exception (please refer to the Data Availability Statement in the manuscript PDF file). The data should be provided as part of the manuscript or its supporting information, or deposited to a public repository. For example, in addition to summary statistics, the data points behind means, medians and variance measures should be available. If there are restrictions on publicly sharing data—e.g. participant privacy or use of data from a third party—those must be specified.

Reviewer #1: Yes

Reviewer #2: Yes

**RESPONSE: Thank you.**

5. Is the manuscript presented in an intelligible fashion and written in standard English?

PLOS ONE does not copyedit accepted manuscripts, so the language in submitted articles must be clear, correct, and unambiguous. Any typographical or grammatical errors should be corrected at revision, so please note any specific errors here.

Reviewer #1: Yes

Reviewer #2: Yes

**RESPONSE: Thank you.**

6. Review Comments to the Author

Please use the space provided to explain your answers to the questions above. You may also include additional comments for the author, including concerns about dual publication, research ethics, or publication ethics. (Please upload your review as an attachment if it exceeds 20,000 characters)

Reviewer #1: The paper has been written well, and it analyzes the patterns, trends, and determinants of OOP expenses for medicines in Bangladeshi households using nation-wide survey data from the HIES. The paper provides a lot of empirical evidence on the subject using appropriate analytical methods. For instance, the paper analyses trends and determinants of OOP household expenses on medicines across different income quintiles.

**RESPONSE: Thank you.**

Please check whether you could attend the followings which may further improve your paper:
• From the view point of public policy, it may be important to discuss about income elasticity of OOP expenses on medicine. Can you calculate the elasticity using double-log regression framework? Analyzing the data from elasticity ground may be more important than analyzing the same across different income quintiles. This type of an analysis may be useful to determine whether medicine is an essential or a luxury for Bangladeshi households.

**RESPONSE: As the reviewer suggests we have incorporated this aspect in the discussion section.**

**“Sixth, this study does not analyze medicines prices or price elasticity as a factor affecting medicines out-of-pocket expenditure because information on medicine prices is not collected as part of the survey. Linking outside data source with the data set used in this study is challenging as individuals in the survey do not report on specific products that they purchased.”**

**Moreover, it is noteworthy to mention to the reviewer that medicine price information collected through market studies often do not reflect the prices patients pay for their medicines.**

• The price-levels of medicines play a significant role in determining the extent of OOP expenses on medicines. Did take price-factor into account when analyzing? If not, the current analysis is lop-sided, and resultantly, the paper is missing a big picture coming from the price factor. You may use the data from price indices of medicine or any other source to capture the impact of prices on OOP expenses on medicines.

**RESPONSE: Thank you for this comment. We have included an explanation in the discussion section about this point. See response to the comment above.**

**It is important to note that the National Household and Expenditure Survey asks the respondents to provide the information on the total costs of their medicines over the past 30 days. It does not ask the respondents about the price of each of the medicine purchased. We do not know how many medicines individuals consumed.**

**It is also important to take into consideration that the amounts analyzed are at current market prices and that there are no reliable price indices for medicines in the different geographical areas, so the price factor mentioned could not be effectively controlled. Furthermore, it is important to mention that correcting for inflation would not modify the results obtained since this survey is cross-sectional and not a comparative one of different points of time.**

• The covariates that the paper considered represent only the demand-side of medicines. The paper misses the story coming from supply-side factors in determining OOP expenses on medicines. Availability of public/private avenues for treatments, proximity to such avenues, health insurance coverage, facilities available in public/private avenues, influence of doctors are some of supply-side factors. Can you strengthen the analysis by incorporating the information pertaining to those supply-side factors? Accordingly, you may strengthen the sections of discussions and conclusions by describing the situation and the role of such supply-side factors in determining OOP expenses on medicines.

**RESPONSE: The reviewer is correct that this analysis focuses on the demand-side. It would be very interesting to link the demand side data with the supply side. However, this is outside the scope of this manuscript. In addition, it is noteworthy, that linking supply side factors such as availability of medicines in public and private sector and prescriber behavior in the catchment area of individual households are often absent or not updated. We have included this limitation in the discussion sector.**

**“Finally, this study focuses on determinants of out-of-pocket expenditure that are recorded within this survey. We did not link other databases to study determinants of expenditure such as distance to the nearest government/private hospital, nearest public/private clinics, nearest public/private dispensary, and availability of doctors, specialists, dentists per 1,000 of population as they might have an explanatory power.”**

Reviewer #2: Thank you for implementing necessary changes. the paper has improved in content and presentation. Limitations of the study may be highlighted.

**RESPONSE: Thank you.**
